# A Mysterious Paratracheal Mass: Pulmonary Agenesis

**DOI:** 10.7759/cureus.8738

**Published:** 2020-06-21

**Authors:** Qian Zhang, Khine S Shan

**Affiliations:** 1 Internal Medicine, Abington Hospital - Jefferson Health, Abington, USA; 2 Internal Medicine, University of Maryland Medical Center, Baltimore, USA

**Keywords:** pulmonary agenesis, tuberculosis, paratracheal mass, tumor

## Abstract

A 35-year-old lady with a history of possible tuberculosis infection 15 years ago presented to the clinic with the chief complaint of cough. Incidental chest CT showed a right paratracheal and medial right apical heterogeneous soft tissue mass with central areas of calcification that warranted further investigation. A routine endobronchial ultrasound-guided transbronchial needle aspiration (EBUS-TBNA) discovered isolated lobar pulmonary agenesis as the underlying cause of the mass without findings of malignancy on pathology reports.

## Introduction

Pulmonary agenesis is a very rare congenital anomaly associated with a lack of proper development from the primary lung bud [1]. It is further classified into different subtypes based on the morphology. Each subtype correlates with different clinical manifestations or outcomes [2]. Our case report details an otherwise healthy 35-year-old lady with reported possible exposure to an active tuberculosis patient 15 years ago that ultimately was found to have a biopsy-proven paratracheal lobar pulmonary agenesis.

## Case presentation

The patient is a 35-year-old lady presented to the primary care physician with the chief complaint of cough. Her past medical history is significant for a possible tuberculosis infection 15 years ago. She was in direct contact with another patient who was positive for tuberculosis at a gathering event and later completed a six-month course of anti-tuberculosis regimen prescribed by her primary care physician. She otherwise remained to be asymptomatic and healthy. Her nonproductive cough began three weeks ago. She denied fever, chest pain, dyspnea, night sweats, and weight loss but did complain of sore throat and occasional chills. She lived at home with her family and reported to have sick contact with her kids as they were suffering from an upper respiratory infection. She decided to present to the office for further evaluation due to concerns about possible tuberculosis reactivation. 

**Figure 1:**
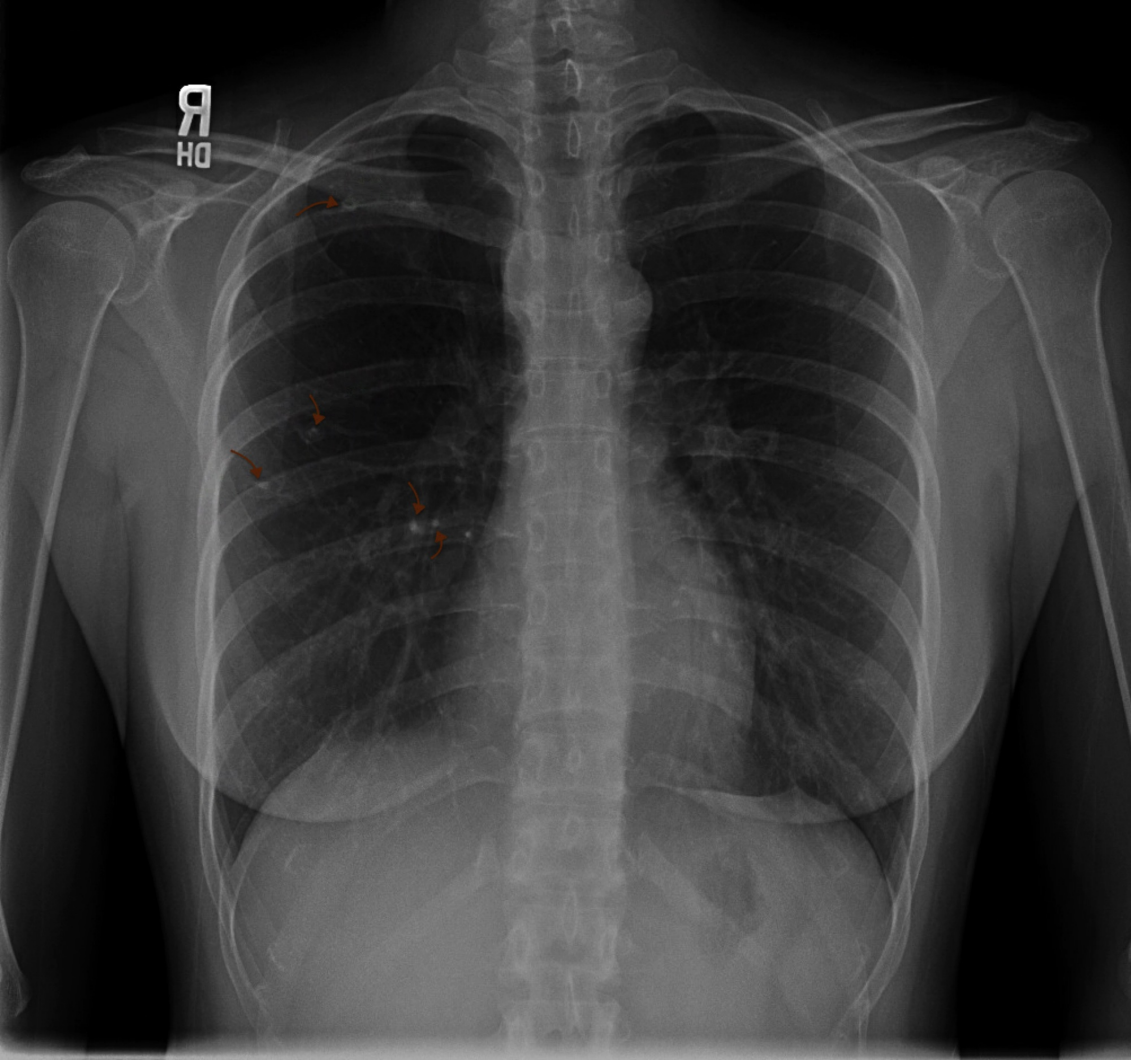
Chest X-Ray There were small rim calcified lesions in the right mid and upper lung zones suggestive of a benign etiology without findings of active or latent tuberculosis infection. R: right side.

Her initial vital signs on presentation were as follows: blood pressure 116/59 mmHg, heart rate 81 beats per minute, temperature 97.8°F, body mass index 20.97. Physical examination was completely benign. There was a low clinical suspicion of active tuberculosis given her overall clinical impression. She was recommended to follow up in the clinic in a few weeks after obtaining a two-view chest x-ray (CXR), sputum acid-fast bacillus (AFB) smear, and culture. The patient was followed up via telemedicine over the next few weeks. She continued to have a mild cough with a low clinical suspicion for tuberculosis. The AFB smear and culture could not have been completed due to a lack of sputum samples. CXR revealed small rim calcified lesions in the right mid and upper lung zones suggestive of a benign etiology without findings of active or latent tuberculosis infection (Figure 1). The patient was recommended to schedule a follow-up appointment in six months while maintaining a close observation of her symptoms. 

**Figure 2:**
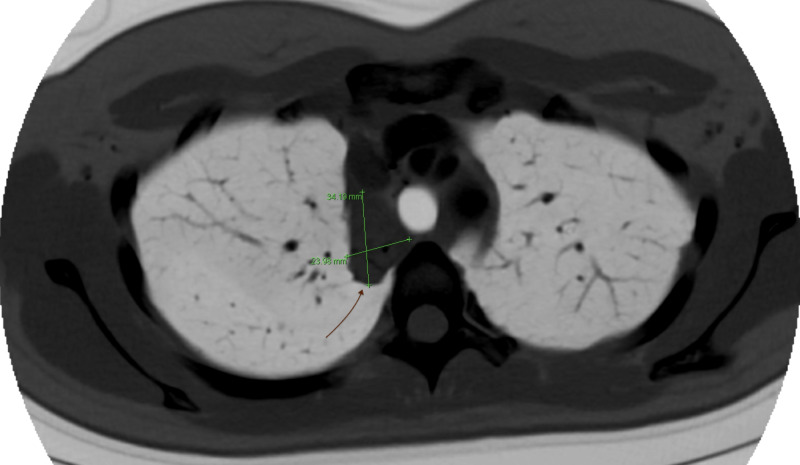
Chest CT With Contrast A high right paratracheal and medial right apical heterogeneous soft tissue with central areas of calcification.

Six months later, her cough reoccurred after an initial improvement over the past few months. The cough was accompanied by occasional sputum. She denied fever, chills, night sweat, sore throat, chest pain, dyspnea, or weight loss. She had been in close contact with people who were diagnosed with an upper respiratory infection. She was recommended to undergo a pulmonary function test (PFT) as well as a chest CT for further evaluation. She was prescribed dextromethorphan for symptomatic relief, educated on the symptoms of active tuberculosis, and instructed to give frequent updates of her symptoms. CT with intravenous contrast revealed a high right paratracheal and medial right apical heterogeneous soft tissue with central areas of calcification (Figure 2). She agreed to opt for a routine endobronchial ultrasound-guided transbronchial needle aspiration (EBUS-TBNA) to rule out malignancy. 

Bronchoscopy inspection revealed unremarkable trachea, left upper lobe, left lingula, left lower lobe, right middle lobe, and right lower lobe; but interestingly, no right upper lobe was observed. Furthermore, EBUS-TBNA showed a right paratracheal mass without surrounding lymphadenopathies that was biopsied and sent to pathology for analysis. The presumptive diagnosis for the right paratracheal mass was likely due to a nondeveloped right upper lung with associated scarring and atelectasis. The pathology report later confirmed the finding as there was no indication of malignancy or fungal infection.

## Discussion

Our patient was an otherwise healthy young lady without malignancy risk factors found to have an incidental finding of a paratracheal mass on CT scan of the chest as she was being worked up for cough. Interestingly, right upper lobe agenesis was discovered during bronchoscopy with associated scarring and atelectasis that was responsible for the mass confirmed by pathology specimens. Pulmonary agenesis is a very rare congenital abnormality that is due to the lack of development and growth of the primary lung bud due to either a primary embryogenic defect or is a consequence of conditions that restricts fetal lung growth [1]. Moreover, pulmonary agenesis is classified by the Spencer classification criteria based on the appearance of the undeveloped lung. It could affect the unilateral side of the lung, bilateral sides of the lung, or could even affect a particular lobe [2]. Prior to the Spencer classification, Schneider originally had classified agenesis into three different groups in 1912 that was later modified by Boyden [3]. Type I or agenesis displayed completely absent lung parenchyma, bronchus, and blood vessels. Type II or aplasia showed a complete absence of lung tissue with a rudimentary bronchus. Lastly, type III or hypoplasia illustrated a variable degree of the lung parenchyma, bronchus, and blood vessels [3]. Despite the exact incident of pulmonary agenesis is unknown, it is reported that more than 50% of the cases are accompanied by other forms of congenital abnormalities, as it could affect normal fetal development that subsequently leads to premature death [1]. A total of fewer than 220 cases of pulmonary agenesis were reported worldwide prior to 1970 [2]. Most of the cases were discovered during infancy or early childhood as subtypes such as the bilateral pulmonary agenesis is incompatible with life. However, isolated lobar agenesis has been rarely reported based on our literature review.

Isolated lobar agenesis could often be asymptomatic. Our patient denied reports of other health issues over the past 35 years despite the exposure with a patient with possible tuberculosis. However, there was no obvious radiographic evidence based on her CXR and CT chest findings of tuberculosis. In addition, most of the patients who were later discovered to have isolated lobar agenesis are often associated with an abnormal CXR that warranted a further detailed workup. Common CXR findings include decreased lung volume, a right shift of mediastinum, or even an elevation of the right hemidiaphragm base [4]. However, a CT chest is the image of choice after an initial undiagnostic CXR [5]. Other imaging modalities, which include bronchography, pulmonary angiography, MRI angiography, are indicated if further extensive workup is clinically necessary. No treatment is indicated in asymptomatic cases of isolated lobar agenesis unless in circumstances where it is the source of recurrent pulmonary infections [6].

Anatomist divided the thorax into three different compartments: anterior, middle, and posterior mediastinum [7]. The trachea is located in the middle mediastinum. The most common lesion found in the middle mediastinum is the bronchogenic cyst that is often located near the tracheal carina (52%) or otherwise in the paratracheal region (19%) [8,9]. It has a CT finding of a homogeneous appearance unlike the heterogeneous soft tissue structure found in our patient. Our patient had a slight concern of possible malignancy given the paratracheal soft tissue mass found on CT scan despite having an overall low clinical suspicion. Thus, EBUS-TBNA was elected to further investigate the exact etiology and confirm a lack of malignancy. EBUS-TBNA is a minimally invasive procedure that allows the interventionist to sample tissues via fine-needle aspiration under the guidance of direct real-time sonographic visualization to further guide the clinical course as well as treatment. It serves as high accuracy and low mortality measurement that is beneficial for patients [10].

## Conclusions

Pulmonary agenesis is a very rare congenital anomaly that is divided into different categories based on its morphology. Patients with lobar agenesis are often asymptomatic and discovered incidentally on CXR or CT scans. EBUS-TBNA could be used to confirm the diagnosis. No treatment or routine follow-up is advised as the condition is benign in nature. 
